# Facilitating accessible, rapid, and appropriate processing of ancient metagenomic data with AMDirT

**DOI:** 10.12688/f1000research.134798.2

**Published:** 2024-05-28

**Authors:** Maxime Borry, Adrian Forsythe, Aida Andrades Valtueña, Alexander Hübner, Anan Ibrahim, Andrea Quagliariello, Anna E. White, Arthur Kocher, Åshild J. Vågene‬, Bjørn Peare Bartholdy, Diāna Spurīte, Gabriel Yaxal Ponce-Soto, Gunnar Neumann, I-Ting Huang, Ian Light, Irina M. Velsko, Iseult Jackson, Jasmin Frangenberg, Javier G. Serrano, Julien Fumey, Kadir T. Özdoğan, Kelly E. Blevins, Kevin G. Daly, Maria Lopopolo, Markella Moraitou, Megan Michel, Meriam van Os, Miriam J. Bravo-Lopez, Mohamed S. Sarhan, Nihan D. Dagtas, Nikolay Oskolkov, Olivia S. Smith, Ophélie Lebrasseur, Piotr Rozwalak, Raphael Eisenhofer, Sally Wasef, Shreya L. Ramachandran, Valentina Vanghi, Christina Warinner, James A. Fellows Yates

**Affiliations:** 1 Cluster of Excellence "Balance of the Microverse", Leibniz Institute for Natural Product Research and Infection Biology Hans Knöll Institute, Adolf-Reichwein-Straße 23, Jena, Thuringia, 07745, Germany; 2 Department of Archaeogenetics, Max Planck Institute for Evolutionary Anthropology, Deutscher Pl. 6, Leipzig, Saxony, 04103, Germany; 3 Department of Animal Zoology, Uppsala Universitet, Norbyvägen 18D, Uppsala, 752 36, Sweden; 4 Associated Research Group of Archaeogenetics, Leibniz Institute for Natural Product Research and Infection Biology Hans Knöll Institute, Adolf-Reichwein-Straße 23, Jena, Thuringia, 07745, Germany; 5 Department of Paleobiotechnology, Leibniz Institute for Natural Product Research and Infection Biology Hans Knöll Institute, Adolf-Reichwein-Straße 23, Jena, Thuringia, 07745, Germany; 6 Department of Comparative Biomedicine and Food Science, Universita degli Studi di Padova, Viale dell'Università 16, Legnaro, Padova, 350250, Italy; 7 Section for Molecular Ecology and Evolution, Globe Institute, Faculty of Health and Medical Sciences, Københavns Universitet, Øster Farimagsgade 5, Copenhagen K, 1353, Denmark; 8 BioArCh, Department of Archaeology, University of York, York, England, YO10 5DD, UK; 9 Transmission, Infection, Diversification and Evolution Group, Max Planck Institute for Geoanthropology, Kahlaische Str. 10, Jena, Thuringia, 07745, Germany; 10 Section for Hologenomics, Globe Institute, Faculty of Health and Medical Sciences, Københavns Universitet, Oester Voldgade 44747, Copenhagen K, 1350, Denmark; 11 Department of Archaeological Sciences, Universiteit Leiden, Einsteinweg 2, Leiden, 2333 CC, The Netherlands; 12 Institute of Ecology and Evolution, Friedrich-Schiller-Universität Jena, Jena, Thuringia, 07743, Germany; 13 Microbial Paleogenomics Unit, Institut Pasteur, Université Paris Cité, CNRS UMR 2000, Rue du Docteur Roux 25-28, Paris, Île-de-France, F-75015, France; 14 Max Planck-Harvard Research Center for the Archaeoscience of the Ancient Mediterranean (MHAAM), Max Planck Institute for Evolutionary Anthropology, Deutscher Pl. 6, Leipzig, Saxony, 04103, Germany; 15 Department of Organismic and Evolutionary Biology, Harvard University, 26 Oxford St., Cambridge, Massachusetts, 02138, USA; 16 Max Planck Institute for Infection Biology, Virchowweg 12, Berlin, Berlin, 10117, Germany; 17 SFI Centre for Research Training in Genomics Data Science, University of Galway, Galway, H91 TK33, Ireland; 18 Smurfit Institute of Genetics, The University of Dublin Trinity College, Dublin, Leinster, D02 VF25, Ireland; 19 Department of Biochemistry, Microbiology, Cell Biology and Genetics, Universidad de La Laguna, San Cristóbal de La Laguna, Santa Cruz de Tenerife, 38200, Spain; 20 Bioinformatics and Biostatistics Hub, Institut Pasteur, Université Paris Cité, Rue du Docteur Roux 25-28, Paris, Île-de-France, F-75015, France; 21 Animal Ecology, Wageningen Environmental Research, P.O box 47, Wageningen, Gelderland, 6700 AA, The Netherlands; 22 Department of History and Art History, Universiteit Utrecht, Drift 6, Utrecht, Utrecht, 3512 BS, The Netherlands; 23 Center for Bioarchaeological Research, Arizona State University, Candy Mall, Tempe, Arizona, 85281, USA; 24 Department of Archaeology, Durham University, South Road, Durham, County Durham, England, DH1 3LE, UK; 25 Institute of Ecology and Evolution, School of Biological Sciences, The University of Edinburgh, Charlotte Auerbach Road, Edinburgh, Scotland, EH9 3FL, UK; 26 Department of Human Evolutionary Biology, Harvard University, Divinity Avenue 11, Cambridge, Massachusetts, 02138, USA; 27 Department of Anatomy, University of Otago, 270 Great King St, Dunedin, Otago, 9016, New Zealand; 28 International Laboratory for Human Genome Research (LIIGH), Universidad Nacional Autonoma de Mexico, La Mesa 3001, Juriquilla, Queretaro, 76230, Mexico; 29 Center for Genomic Sciences (CCG), Universidad Nacional Autonoma de Mexico, Cuernavaca, Morelos, 62210, Mexico; 30 Institute for Mummy Studies, Eurac Research, Drususallee 1, Bolzano/Bozen, Autonome Provinz Bozen, 39100, Italy; 31 Centre for Integrative Biology (CIBIO), Universita degli Studi di Trento, Via Sommarive 9, Povo, Trentino, 38123, Italy; 32 Department of Anatomy and Anthropology and Department of Human Molecular Genetics and Biochemistry, Faculty of Medicine, Tel Aviv University, Ramat Aviv, Tel Aviv-Yafo, 69978, Israel; 33 National Bioinformatics Infrastructure Sweden, Science for Life Laboratory, Tomtebodavägen 23, Stockholm, 17165, Sweden; 34 Department of Biology, Lunds Universitet, Sölvegatan 35, Lund, 223 62, Sweden; 35 Department of Integrative Biology, The University of Texas at Austin, Speedway 2415, Austin, Texas, 78712, USA; 36 Instituto Nacional de Antropología y Pensamiento Latinoamericano, 3 de Febrero 1370 (1426), Ciudad Autónoma de Buenos Aires, C1426BJN CABA, Argentina; 37 Center for Anthropobiology and Genomics of Toulouse, CNRS/Universite Toulouse III Paul Sabatier, Allées Jules Guesde 37, Toulouse, Occitanie, 31000, France; 38 Department of Computational Biology, Adam Mickiewicz University, Poznań, Uniwersytetu Poznanskiego 6, Poznań, Wielkopolska, 61-614, Poland; 39 Center for Evolutionary Hologenomics, Globe Institute, Københavns Universitet, ester Voldgade 44747, Copenhagen, Copenhagen K, 1350, Denmark; 40 Defence Genomics, Centre for Genomics and Personalised Health, Queensland University of Technology, Musk Ave 60, Kelvin Grove, Queensland, 4059, Australia; 41 Department of Human Genetics, The University of Chicago, E. 58th St. 920, Chicago, Illinois, 60637, USA; 42 Department of Anthropology, Harvard University, Divinity Avenue 11, Cambridge, Massachusetts, 02138, USA; 43 Faculty of Biological Sciences, Institute of Microbiology, Friedrich-Schiller-Universität Jena, Neugasse 25, Jena, Thuringia, 07743, Germany

**Keywords:** metagenomics, environmental, palaeogenomics, aDNA, microbiome, metadata, microbial, FAIR data

## Abstract

**Background:**

Access to sample-level metadata is important when selecting public metagenomic sequencing datasets for reuse in new biological analyses. The Standards, Precautions, and Advances in Ancient Metagenomics community (SPAAM, https://spaam-community.org) has previously published AncientMetagenomeDir, a collection of curated and standardised sample metadata tables for metagenomic and microbial genome datasets generated from ancient samples. However, while sample-level information is useful for identifying relevant samples for inclusion in new projects, Next Generation Sequencing (NGS) library construction and sequencing metadata are also essential for appropriately reprocessing ancient metagenomic data. Currently, recovering information for downloading and preparing such data is difficult when laboratory and bioinformatic metadata is heterogeneously recorded in prose-based publications.

**Methods:**

Through a series of community-based hackathon events, AncientMetagenomeDir was updated to provide standardised library-level metadata of existing and new ancient metagenomic samples. In tandem, the companion tool 'AMDirT' was developed to facilitate rapid data filtering and downloading of ancient metagenomic data, as well as improving automated metadata curation and validation for AncientMetagenomeDir.

**Results:**

AncientMetagenomeDir was extended to include standardised metadata of over 6000 ancient metagenomic libraries. The companion tool 'AMDirT' provides both graphical- and command-line interface based access to such metadata for users from a wide range of computational backgrounds. We also report on errors with metadata reporting that appear to commonly occur during data upload and provide suggestions on how to improve the quality of data sharing by the community.

**Conclusions:**

Together, both standardised metadata reporting and tooling will help towards easier incorporation and reuse of public ancient metagenomic datasets into future analyses.

## Introduction

The field of palaeogenomics has been praised as a role model for scientific data reporting and data availability. ^1^ When compared against FAIR principles (Findability, Accessibility, Interoperability, and Reusability), ^2^ ancient DNA (aDNA) sequencing data have been consistently made available in standard data formats on public data repositories, satisfying the principles of *accessibility*, *interoperability* and, to a certain extent, *reusability.* However, the *findability* of the uploaded data still poses challenges, often due to the lack of inclusion of key metadata specific for aDNA in the standardised sample metadata fields used by public sequencing repositories such as the European Bioinformatic Institute’s European Nucleotide Archive (EBI ENA), the US National Center for Biotechnology Information’s Sequence Read Archive (NCBI SRA), and the Japanese National Institute of Genetics’ DNA Data Bank of Japan (NIG DDBJ). To improve findability of ancient metagenomic samples in public data repositories, the SPAAM community ( https://spaam-community.org) previously developed the AncientMetagenomeDir project, a set of curated standard sample metadata for ancient host-associated shotgun-sequenced metagenomes, ancient environmental metagenomes, and/or host-associated microbial genomes. ^3^ However, while sample-level metadata already help with the discovery of suitable comparative data, library-level metadata are also needed to further facilitate data reuse in dedicated aDNA analysis pipelines such as PALEOMIX, ^4^ nf-core/eager, ^5^ aMeta, ^6^ and nf-core/mag. ^7^ aDNA researchers often build many different types of NGS libraries ^8^ and may generate (meta)genomic data using multiple different sequencing platforms that require different bioinformatic pre-processing workflows. Furthermore, the library-level metadata currently available in public repositories often lack key information about aDNA library treatments and other laboratory information needed to reproducibly reanalyse palaeogenomic datasets obtained from different studies.

**Figure 1.**
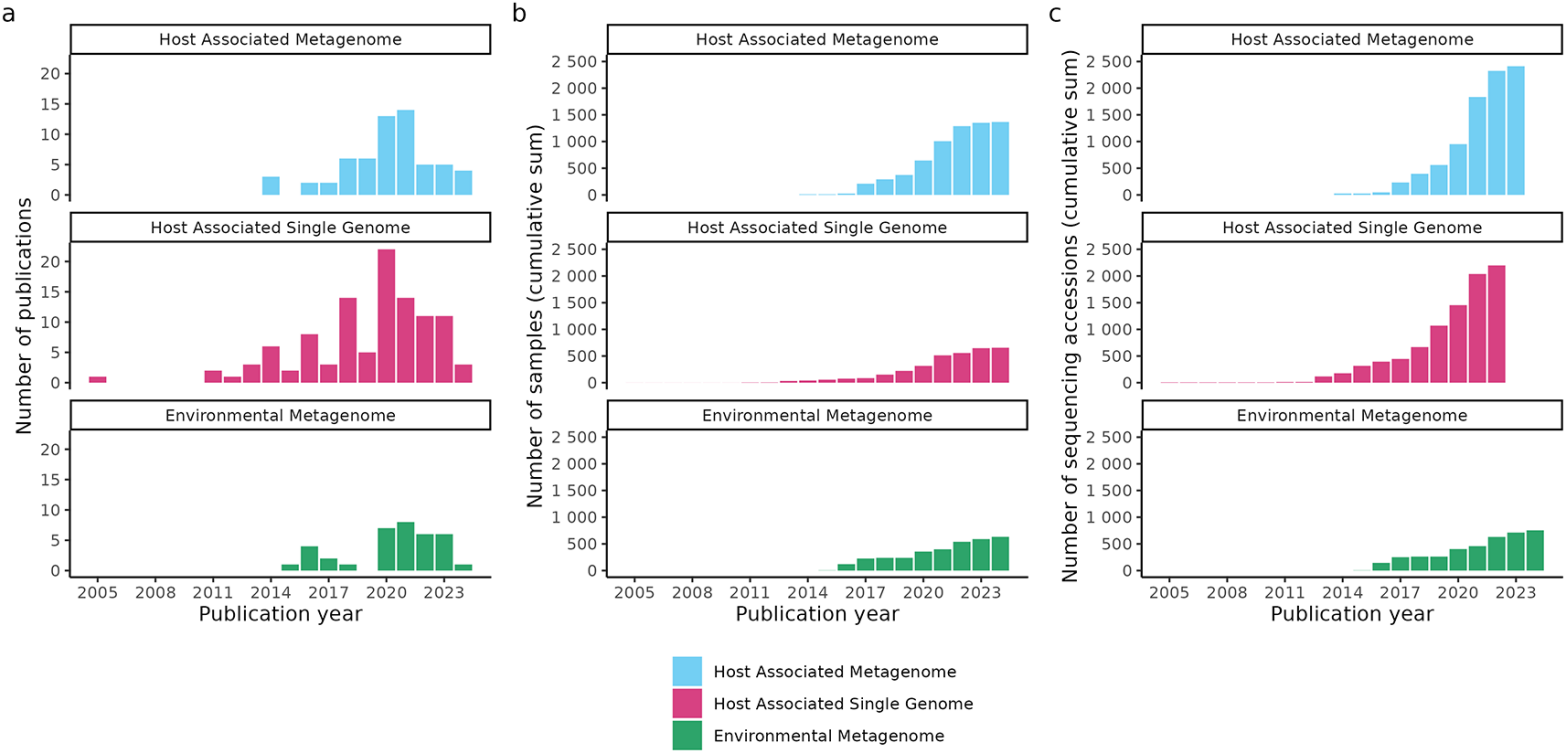
Growth of studies curated in the AncientMetagenomeDir as of v24.03. (a) Number of ancient metagenomic publications published per year with open sequencing data and included in AncientMetagenomeDir. The original AncientMetagenomeDir publication was in 2020. (b) Cumulative sum of the number of published samples with publicly accessible sequencing data. (c) Cumulative sum of the number of ancient metagenomic sequencing data accessions of the samples in panel b. Data from Fellows Yates et al. ^12^

An ancient metagenome can be generally described as the entire genetic content of a sample, within which at least a portion of the DNA has degraded over time. ^3^ As the number of ancient metagenomics samples and shotgun sequenced library files steadily increases (currently >2500 host-associated metagenome, >3000 single-genome, and >700 environmental metagenome sequencing run accessions as of April 2024; Figure 1), the need to efficiently identify, curate, and download such data is becoming more pressing. Although the original AncientMetagenomeDir releases provided project- or sample-level accession numbers that point to data primarily hosted by the ENA, SRA, and DDBJ, the metadata tables did not provide direct links to the data themselves. This meant that researchers still needed to manually search for each project or sample accession number in public data repositories and then manually identify and download the relevant associated files. Researchers were then required to parse and evaluate each sequencing file for inclusion in their study by consulting the original scientific publications for laboratory, library, and sequencing metadata. As with sample metadata, the reporting of this information within publications can be heterogeneous, may appear in the main text or supplement, and may take the form of prose text, tables, supplementary spreadsheets, or citations to other publications or protocols. While other tools for exploring public data repositories exist, such as NCBImeta, ^9^ SRA-Explorer, ^10^ and ffq, ^11^ they are generally limited to a restricted set of metadata available for inspection or require the use of command-line filtering tools, an interface not always accessible to all palaeogenomics researchers, who often have varying levels of computational experience.

Here we present AMDirT ( **A**ncient **M**etagenome **Dir T**oolkit), a tool designed to assist researchers in using a new extension of AncientMetagenomeDir that now includes aDNA library- and sequencing-level metadata. AMDirT is designed to provide a solution to four different challenges, thanks to new command line interfaces (CLI), a new graphical user interface (GUI), and a hosted web version ( https://spaam-community.org/AMDirT). First, new CLI tooling helps contributors to AncientMetagenomeDir to curate newly published aDNA sequencing data in AncientMetagenomeDir by automatically retrieving relevant library-level metadata available from sequencing archives ( autofill CLI command) and preparing semi-filled data entry tables for submission. Second, AMDirT also helps project reviewers automate a variety of data validation tasks on completed entry tables to ensure consistency ( validate CLI command, an improved version of the AncientMetagenomeDirCheck tool from Ref. 3). Third, AMDirT now provides users with a web browser-based GUI that allows researchers to explore relevant ancient metagenomics-related sequencing datasets in AncientMetagenomeDir tables ( viewer command) and CLI interfaces ( download and convert CLI commands) to download metadata and export data download scripts from International Nucleotide Sequence Database Collaboration (INSDC) repositories. Finally, as an additional functionality, both AMDirT viewer GUI and CLI interfaces can generate template input configuration files for a suite of standard aDNA metagenomics-related pipelines in order to further automate and accelerate the processing of such aDNA data. AMDirT is available for installation via PyPI ( https://pypi.org/) or Bioconda, ^13^ with source code on GitHub under https://github.com/SPAAM-community/AMDirT.

## Methods

### Implementation

**AMDirT tool implementation**. Members of the SPAAM community an international and open community of nearly 500 researchers work on ancient metagenomics ( https://spaam-community.org), developed AMDirT through a series of code sprints and hackathons, using Python (v3.9, https://www.python.org/; RRID:SCR_008394). It is accessible via a command-line-interface written using Click or via a python API ( https://click.palletsprojects.com/). The autofill command uses the ENA portal API ( https://www.ebi.ac.uk/ena/portal/api/) ^14^ to automatically query and return metadata associated with the sequencing library level, such as all project, samples and library accessions, location and size of FASTQ files, sequencing instrument model, library strategy and layout, as well as read count. Data validation in the validate subcommand is performed using the jsonschema python library ( https://python-jsonschema.readthedocs.io/) by validating the dataframes containing the sample level and library level metadata against their respective JSON schema, and using a variety of checks written using Pandas ^15^ to avoid data duplication and ensure consistency of new entries. Additionally, validate will also check that each publication has its own valid DOI, that each sequence archive accession is valid, unique, and associated with the correct project accession. Any errors will be reported in table format, indicating the type of error, the line and column location of the error, and a short explanation of the error, and how to fix it. Both tools are primarily used within automated GitHub actions processes on the AncientMetagenomeDir GitHub repository, however are also usable by submitters and curators running on their own machines.

The GUI data exploration interface of the viewer command was developed using Streamlit ( https://streamlit.io/), and the streamlit-aggrid library ^16^ is used to allow the end-user to interactively filter and prepare configuration files to process ancient (meta)genomic data in bioinformatics pipelines. AMDirT is packaged thanks to setuptools, ^17^ and is distributed on PyPi and Bioconda. ^13^ The source code is available on GitHub ( github.com/SPAAM-community/AMDirT), and associated documentation is provided online ( amdirt.readthedocs.io). Furthermore, an online serverless version of the AMDirT viewer tool is available at https://spaam-community.org/AMDirT thanks to the stlite library ( https://github.com/whitphx/stlite), a port of streamlit to WebAssembly that is supported (at the time of writing) by most Chrome-based browsers.

The CLI based convert command reuses the backend of the viewer command to provide a terminal based filtering functionality for more advanced users. Finally, the download command provides a CLI interface to download the different AncientMetagenomeDir tables using the standard python library, with the possibility of specifying the release and the table type.

**AncientMetagenomeDir library metadata aggregation**. To extend the original AncientMetagenomeDir ^3^ repository to include library metadata, we created new tab-separated value (TSV) tables and their associated validation checks in the form of JSON schema files, following the original AncientMetagenomeDir structure.

We retained the TSV format for maximum software compatibility, as originally described in Ref. 3. Fields included in the new library-level schema were selected after consultation with ancient metagenomics researchers of the SPAAM community, and, where relevant and possible, by mirroring existing metadata fields and controlled vocabulary from the ENA repository. Newly added library information columns include the library name (how data are typically reported in original publications), the aDNA library generation method (e.g., double-stranded or single-stranded libraries), the library indexing polymerase (e.g., proof-reading or non-proofreading), and the library pretreatment method (e.g., non-Uracil-DNA Glycosylase (UDG), full-UDG, or half-UDG treatments). The latter three fields represent information about the sequencing library construction that influences the presence of aDNA damage, a factor that is critical for the processing of aDNA NGS data. ^8^^,^^18^ Sequencing metadata columns include instrument model, library layout (single- or paired-end), library strategy (whole genome sequencing, targeted capture, etc.), and read count. These metadata are also critical for correct processing of aDNA data. For example, whether an instrument uses 2- or 4-colour sequencing chemistry determines if poly-G tail trimming is required to remove sequence artefacts that arise in aDNA reads that are normally shorter than the number of sequencing cycles. Library layout is also necessary to indicate whether read-pair merging needs to be applied prior to mapping, or whether unmerged read pairs are available for *de novo* assembly. The remaining columns provide information about storage and file retrieval of sequencing data: direct URLs to FASTQ files, ‘md5 checksum’ strings (for post-download integrity verification), and download sizes (for storage space usage estimation). Tables may also be extended to contain field-specific metadata useful for data processing under specific conditions. For new non-ENA/SRA supported fields, such as library polymerase or library treatment, we defined fixed lists via new JSON-based ‘enum’ files stored in the AncientMetagenomeDir repository, as with the sample-level metadata.

Via a series of community events, we then manually carried out data entry and curation for the new columns of metadata by comparing the ENA stored metadata with the methods descriptions in original publications. This procedure identified multiple instances of inconsistencies between the two sources, as well as incorrectly uploaded data and metadata in previously published articles. We describe some of the common issues we encountered in the Discussion section below. In cases of conflict between the publication and the ENA metadata, we attempted to contact the original authors of the publication for confirmation. When this was not possible, we used ‘unknown’ or another missing-data value to indicate uncertainty. Each library-level metadata addition underwent automated validation and peer-review following the same procedure described in Ref. 3. Since the community events, the AMDirT autofill command has been developed to improve the library metadata submission experience by community members contributing new metadata ( Figure 3). The autofill sub-command automates the pulling of ENA metadata into a ‘draft’ library-table format during the continuous integration tests (CI) of a GitHub pull request of sample-level metadata, replacing and improving upon the manually executed R scripts used in the initial pull-down for the community events. Submitters to AncientMetagenomeDir can then copy over much of the metadata and fill in the remaining missing metadata not covered by the existing ENA metadata fields.

**Figure 2.**
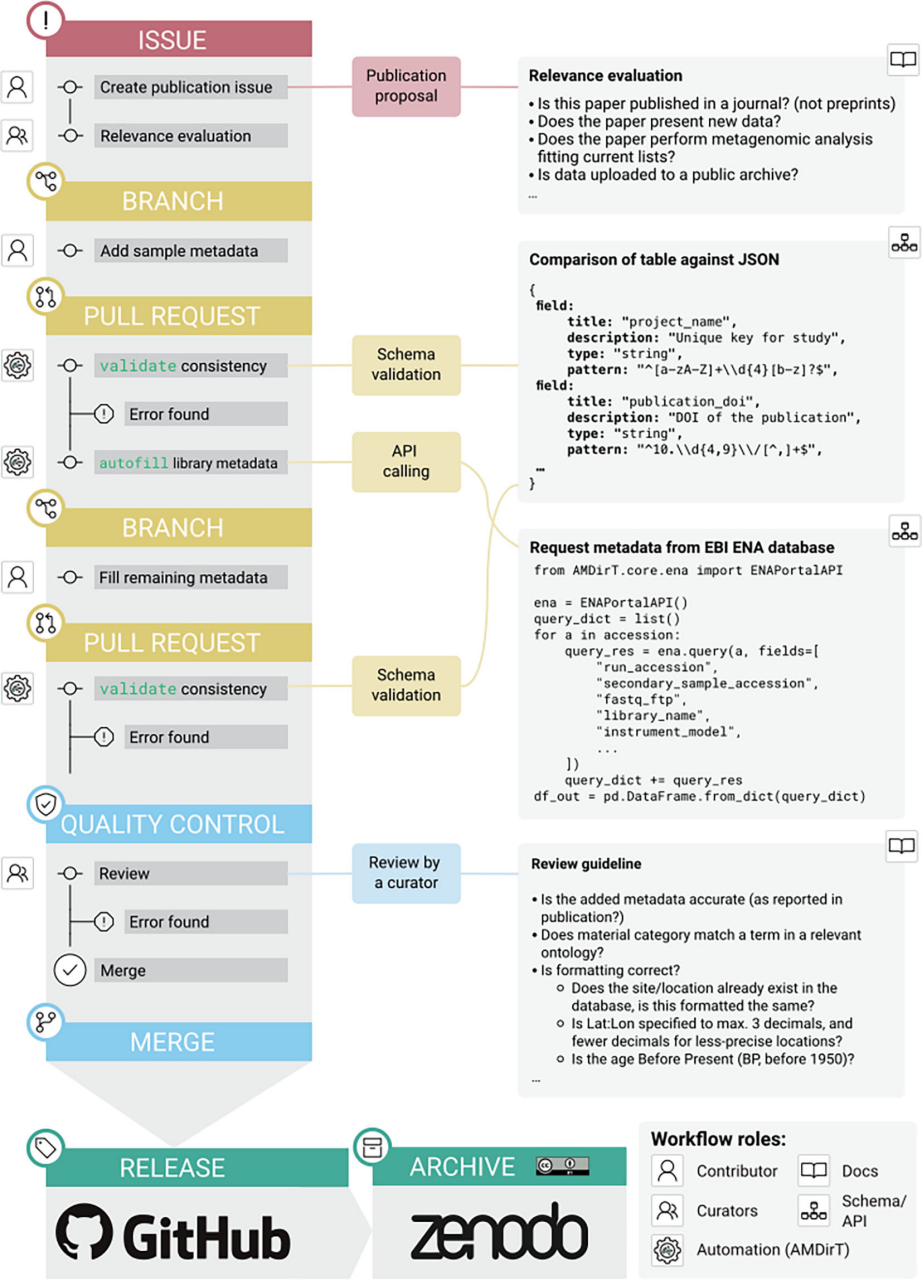
Updated workflow for submission to AncientMetagenomeDir using the AMDirT autofill functionality. The AncientMetagenomeDir submission workflow, as updated since. ^3^ The general workflow remains the same, with issue creation for publication proposals, metadata submission by contributors via a branch and pull request, something that undergoes automated validation (with AMDirT validate), and later peer-review by AncientMetagenomeDir curators. The new addition is the use of autofill that is called via a GitHub Actions ‘bot’. This generates and uploads to the pull request in a comment a partially completed library metadata table that can be filled in, reviewed for accuracy and appended to the corresponding AncientMetagenomeDir library table as a part of the original sample pull request.

### Operation

AMDirT requires a UNIX-based terminal (e.g., Linux, OSX, Windows Subshell for Linux) for both installation and initial usage; however, the toolkit is written in Python and can therefore be used on a wide range of platforms and operating systems.

To install, users are recommended to use the pip or conda package managers. Users who wish to use the GUI based table viewer and downloader will also require any modern web browser supported by Streamlit ( https://streamlit.io/).

For example, to install and load the help message:

$ pip install amdirt



or via conda in a dedicated environment

$ conda create -n amdirt -c bioconda amdirt



The general help of AMDirT (v1.6) is available from the CLI:

 $ AMDirT --help
 Usage: AMDirT [OPTIONS] COMMAND [ARGS]…

   AMDirT: Performs validity check of AncientMetagenomeDir datasets
   Authors: AMDirT development team and the SPAAM community
   Homepage & Documentation: https://github.com/SPAAM-community/AMDirT

 Options:
   --version Show the version and exit.
   --verbose Verbose mode
   --help   Show this message and exit.

 Commands:
   autofill Autofills library and/or sample table(s) using ENA API and…
   convert Converts filtered samples and libraries tables to eager,…
   download Download a table from the AMDirT repository
   merge   Merges new dataset with existing table
   validate Run validity check of AncientMetagenomeDir datasets…
   viewer  Launch interactive filtering tool



Most tools follow a standard CLI based interface. For example, converting a user-filtered ancient metagenome host-associated AncientMetagenomeDir table (e.g. in R) to a curl download script can be performed as follows:

$ AMDirT convert --curl <filtered_table>.tsv ancientmetagenome-hostassociated -o ./



In the command above, options, input files and output files are defined with standard command line flags and positional arguments.

The resulting file AncientMetagenomeDir_curl_download_script.sh file from the command above will be present in the directory specified in the command. The user can then simply run the bash script to download all libraries of the samples present in the input table.

$ bash AncientMetagenomeDir_curl_download_script.sh



For the template pipeline input sheets, these can be supplied to the pipelines themselves, after checking for accuracy.

The other AMDirT tools follow a similar scheme, with help messages and documentation on the AMDirT website providing more how-to information ( https://amdirt.readthedocs.io/).

For the GUI-based viewer tool, a user simply enters the following command in their terminal, after which their web browser will automatically load. Alternatively, the reported local or network address can be manually entered into the user’s web browser. In comparison to the convert subcommand, the input tables are automatically pulled from the AncientMetagenomeDir for the user, without requiring any manual input.

$ AMDirT viewer
AMDirT [INFO]:
[AMDirT] To close app, press on your keyboard: ctrl+c

  You can now view your Streamlit app in your browser.

  Local URL: http://localhost:8501
  Network URL: http://172.16.9.75:8501



Once completed, the user can close the tab and cancel the command in their terminal (e.g., with ctrl + c).

Alternatively, for individuals who wish to use the viewer but do not wish to deal with software installation and/or are not comfortable with command line interfaces, a hosted online version of the AMDirT viewer is available at https://spaam-community.org/AMDirT accessible with a web browser.

## Use cases

Here we will describe a common use case for when users may wish to use the AMDirT package, namely filtering for a particular subset of metagenomic aDNA data, downloading the resulting data, generating a corresponding semi-prepared input sheet for nf-core/eager, ^5^ and creating a citations file. Full tutorials in text and video format for the following and other AMDirT commands can be found on the AMDirT website ( https://amdirt.readthedocs.io/).

This example scenario demonstrates how a user can download all publicly available ancient host-associated metagenomes published since 2020 from samples originating from Spain using the AMDirT GUI interface. In this hypothetical example, a user may wish to compare the microbial taxonomic profiles of archaeological dental calculus and other skeletal elements in Spain at different time points. In order to distinguish modern and aDNA, the user will likely want to examine the DNA for evidence of chemical degradation, which can be used to authenticate aDNA. To do this, the user will already have selected their preferred dedicated aDNA analysis workflow, such as nf-core/eager, that integrates DNA damage analysis into its pipeline. nf-core/eager requires an input ‘sample sheet’ that describes whether a particular sample has been sequenced over multiple lanes or libraries, and whether aDNA damage has been already removed during laboratory processing. We will show how AMDirT can assist with the creation of this sample sheet with the desired dataset.

This example assumes that the user has already installed AMDirT and nf-core/eager, and has downloaded a *Homo sapiens sapiens* reference genome for host DNA removal.

To load the GUI based viewer and downloading tool, a user enters the following command into their terminal:

$ AMDirT viewer



**Figure 3.**
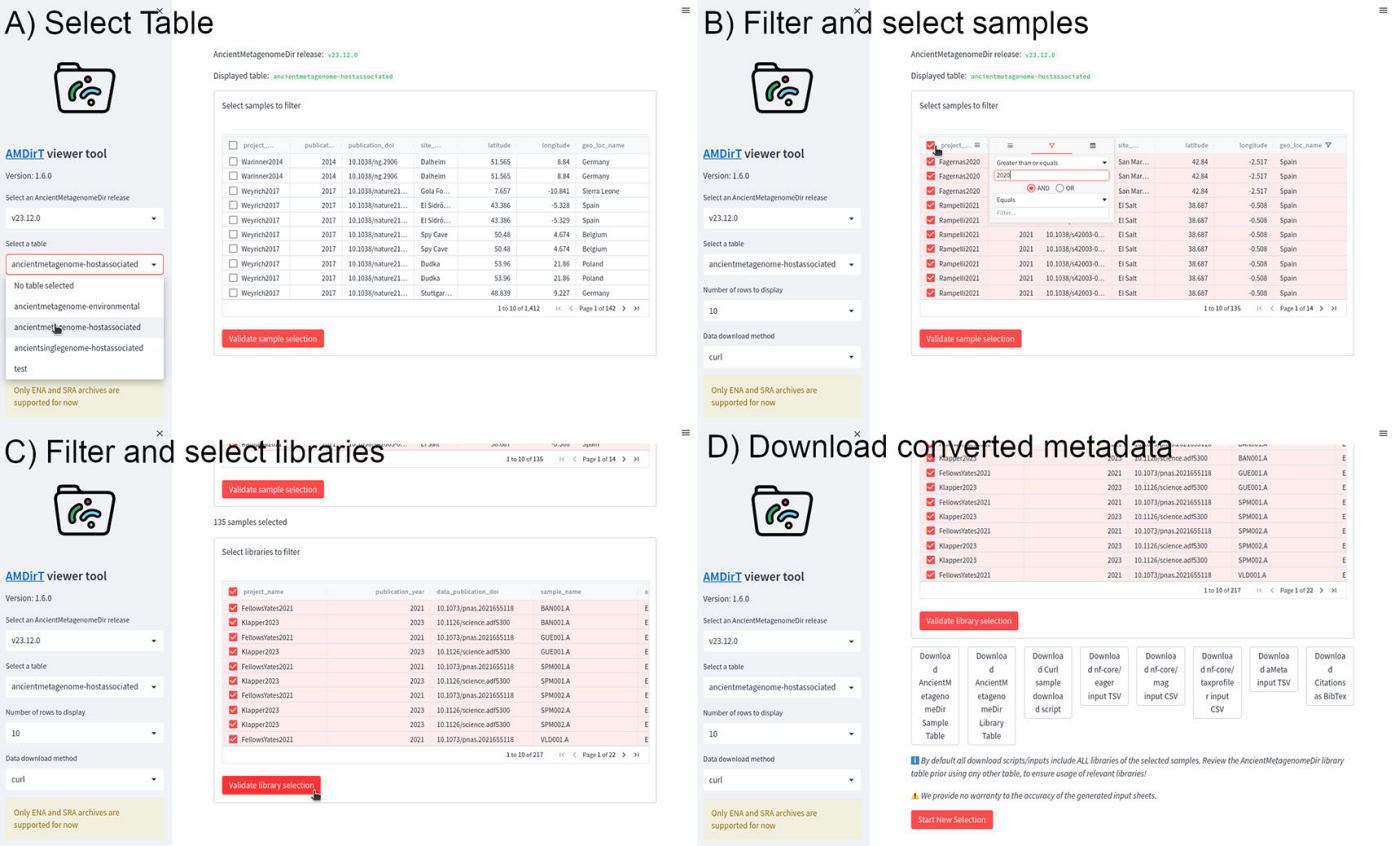
Example workflow of using AMDirT viewer. (a) The viewer opens in a user’s web browser, where the desired AncientMetagenomeDir version and table is selected. (b) Interaction with columns follows standard operations common to most spreadsheet software. Samples for download are selected using checkboxes. (c) The same interface can be used for the subsequent library metadata filter table. (d) After pressing ‘Validate library selection’, buttons appear for downloading various download scripts, reference, and pipeline input sheets.

As shown in Figure 3a, the viewer is loaded into the user’s web browser. Using the left sidebar, the user can navigate drop-down menus to select the release of AncientMetagenomeDir to use (for reproducibility purposes), and the desired AncientMetagenomeDir table to explore (i.e., ancient environmental metagenomes, ancient host-associated metagenomes, or ancient microbial single-genomes). The user can also specify the number of rows for the table to display and which tool to use for the download scripts generated later in the example.

Once the AncientMetagenomeDir table is selected, the main window will load the corresponding table. The user can manipulate the columns as customary in most spreadsheet software, such as resizing the columns by dragging the bars between each column, dragging column names to reorder them, etc. Users can use keyboard arrows or scroll bars to navigate further along the columns. To filter the columns, the user can press the ‘hamburger’ menu of each column, which reveals a range of column operation options. In the example in Figure 3b, the ‘geo_loc_name’ column has already been filtered to display only samples with the value ‘Spain’, and by pressing the funnel on an integer column, such as ‘publication_year’, the user can specify to only display rows ‘greater than or equals’ to 2020. Additional filter specifications can be added using the AND/OR operators in each filter menu. The user can then select which samples to be exported from AMDirT. They can either use the ‘Select All’ checkbox in the top left of the table, or select each sample using the checkboxes at the beginning of each row on a sample-by-sample basis.

Once the user is satisfied with their selection, they can press ‘Validate sample selection’ ( Figure 3c). Once pressed, a new table will appear below the Sample metadata table. This new table contains all library-level metadata of the selected samples from the previous step. Users can then filter library-level metadata with the same interface and in the same way as the sample-level metadata. Once selected, and the ‘Validate library selection’ button has been pressed, a range of buttons representing different downloading options will appear.

Here, we recommend that users always download the corresponding AncientMetagenomeDir libraries table, as well as the BibTeX citation file. In this example, the user would also download the ‘curl download script’ and the ‘nf-core/eager’ input TSV using the corresponding buttons. Hovering over the download script button also provides an estimate for the user of how much hard-drive space the download of all selected data will use.

To close the GUI viewer, the user can close the tab in their browser, and then in their terminal press ctrl + c on their keyboard to stop the Streamlit server.

Alternatively, a user could also follow the same process but via the command line interface. The user first can run the new download ( download) command to retrieve a particular samples or libraries table.

$ AMDirT download --table ancientmetagenome-hostassociated --table_type samples --release v24.03.0



The user could then use typical shell commands, or load the table into languages such as R or Python and Pandas for filtering the table to the data they would be interested in. To replicate the same conditions as in Figure 3b above, a user could run the following command with the common CLI tool awk

$ awk -F "\t" 'NR==1 || $2 >= 2020 && $7 == "Spain"' ancientmetagenome-hostassociated_samples_v24.03.0.tsv > ancientmetagenome-hostassociated_samples_gt2020_spain.tsv



Finally, the user can pass the resulting filtered table to the AMDirT convert command, to perform the same download script generation, pipeline-input file conversion, and citation file download as with the GUI interface

$ AMDirT convert --librarymetadata --curl --eager --bibliography ancientmetagenome-hostassociated_samples_gt2020_spain.tsv ancientmetagenome-hostassociated



The convert command will then generate the same files downloaded by the GUI (example above).

Once downloaded, the user should check the AncientMetagenomeDir libraries table to ensure that all desired libraries are present. If there are extra libraries with specifications that the user does not want, they should remove those entries from the curl download script and nf-core/eager input TSV files. For the CLI convert command, a user can also optionally supply a pre-filtered *libraries* table in addition to the samples table to reduce the need for manual editing of the downloaded files. The user should also review the generated nf-core/eager pipeline input sheet to check for accuracy in regards to the pipeline’s specifications.

After reviewing and filtering the scripts and pipeline input TSV sheets, the user can then use their terminal to navigate to a directory, move the curl script into it and begin the download. Due to the large sizes of sequencing data, in most cases we recommend that a user do this in a ‘screen’ or ‘tmux’ session (or similar) to ensure that the downloading can continue in the background:

$ bash AncientMetagenomeDir_curl_download_script.sh



Once the sequencing data are downloaded, the user can provide their AMDirT generated nf-core/eager input sheet to the following Nextflow ^19^ command (the nf-core/eager input sheet assumes that the command is being run in the same directory as the downloaded data):

$ nextflow run nf-core/eager -r 2.4.6 -profile conda
--input AncientMetagenomeDir_nf_core_eager_input_table.tsv
--fasta hg19.fasta --outdir ./results



## Discussion

### Results of library level metadata aggregation

Since the original publication of AncientMetagenomeDir ^3^ and the release of version v20.09, the SPAAM community has doubled the number of manually curated publications in the AncientMetagenomeDir from 87 to 187 studies as of version v24.03. The number of samples has increased from 443 to 1427 for ancient host-associated metagenome samples, 269 to 667 for ancient microbial genome level sequences, and 312 to 662 for sediment samples ( Figure 1).

During the series of ‘hackathon’ events carried out by the community to scrape library metadata from previous publications and subsequent submissions of new studies, a total of 2557 ancient host-associated metagenome libraries, 3048 ancient microbial genomes libraries, and 754 ancient environmental metagenome libraries have been curated and included.

### Common issues in ancient metagenomic library metadata

During the aggregation and clean-up of the library metadata by the SPAAM community, a range of problems were repeatedly encountered across multiple studies that made data entry and the determination of appropriate preprocessing procedures difficult. Here, we describe the most common issues encountered, as well as possible solutions, listed from most to least severe. By highlighting these common mistakes and problems, we hope to help improve (meta)data uploads to sequencing archives, which in turn will both benefit the AncientMetagenomeDir users, but also the field as a whole.

**Inconsistent sample and library naming.**

*Problem:* A common problem encountered when cross-referencing ENA or SRA metadata with information provided in original publications was inconsistencies in sample, library, and/or sequencing file names. This often made it difficult for the community member to correctly infer which library was associated with which sample, or even which sequencing file went with which library.

*Example:* In studies where two sets of libraries were generated (for example, one with UDG-based DNA damage removal and one without), this palaeogenomic-specific information was often not indicated in the library or file names. Given that this information is not supported in the ENA/SRA metadata schema, this is the only location where such aDNA-specific information could be reasonably recorded. In such cases, we found that while library pretreatment procedures were documented in the original publication, the uploaded metadata and sequencing files generally lacked this information and in some instances used internal laboratory IDs instead of the sample or library codes recorded in the publication. Without a key linking the published IDs with the internal laboratory IDs, other researchers cannot know which files to use for their particular analyses or how to process the data appropriately, and this can lead to downstream problems. For example, if a user does not know that a sequencing file was generated from damage-removed libraries, they may inappropriately apply additional *in silico* trimming steps to remove DNA damage, and thus unnecessarily truncate the sequences.

*Solutions:* We suggest two solutions: first, ancient metagenomic researchers should ensure that library and sample names are descriptive (i.e., in a structured system in which a certain level of information can be inferred just by the name) and that sequencing metadata uploads match those reported in the publication; second, where this is not possible (e.g., if an upload is carried out by a third party), then researchers should at a minimum include a key in their supplementary files. This could be in the form of a table that includes all ID codes for each sample, library, and sequencing batch, including internal laboratory codes, other-analysis codes, and external sequencing archive accession codes.

**Metadata discrepancies about sequencing methods.**

*Problem:* Another relatively common issue was the discrepancies between the metadata reported in the sequence archive and in the original publication. It was generally difficult to resolve such discrepancies without contacting the authors. Discrepancies occurred most frequently in the reporting of the sequencing platform.

*Example:* In several cases, the particular sequencing platform recorded in the sequence archive metadata, such as ‘Illumina HiSeq 4000’, did not match that reported in the publication, e.g. ‘NextSeq 500’.

*Solutions:* Researchers should be sure to cross-reference their metadata upload sheets with their manuscripts prior to upload. In cases where Illumina sequencing was carried out externally (and where limited information may have been provided by the sequencing centre), researchers can generally inspect the headers of the FASTQ file to determine which platform was used, as in the example provided here.

**Methods description in secondary or tertiary citations.**

*Problem:* For journals with strict word or character count limits, it was qualitatively observed that there was an increased tendency in these publications to rely on secondary or tertiary non-protocol specific citations for describing laboratory methods used for DNA library construction and sequencing. This practice is problematic as secondary or tertiary citations may describe multiple protocols, and it was not always possible to determine which protocol was actually used in the study.

*Example:* In one case, a publication reporting an ancient microbial genome reconstruction referred to library protocols used in an earlier related publication that described data generation for a human population genetics study. However, upon closer inspection, this cited study itself referred to an even earlier publication that included extensive protocol experimentation and development. Neither the primary nor secondary publication indicated which of the experimental protocols from the original methods study was actually used.

*Solutions:* Ancient metagenomic researchers should make an effort to more clearly describe their protocols and explicitly indicate which library protocol is linked to each sequencing file. At a minimum, the information provided should include critical metadata for downstream analysis, such as library treatment protocols that affect DNA damage. This can be accomplished by providing expanded, plain-language descriptions of laboratory methods in article supplementary information files (rather than simply citing and re-citing) and providing a supplementary table that lists each library name and their corresponding treatments. For improved compliance with FAIR principles, researchers are encouraged to further provide or cite a protocol written up in a citable protocol format and/or on open platforms. For example, platforms such as protocols.io ^20^ allow critical protocol information to be communicated consistently and unambiguously, by providing a persistent identifier (DOI) that points to a specific version of a given protocol.

**Uploading of mapped BAM files or merged FASTQ files rather than raw metagenomic data.**

*Problem:* Occasionally, we found that in some cases ancient metagenomic researchers uploaded mapped BAM files or merged FASTQ files rather than ‘raw’ FASTQ files (i.e., against ENA/SRA specifications). Both formats present obstacles for downstream analysis. For example, mapped BAM files include only reads mapped to a particular reference genome and thus do not represent a full metagenomic dataset. For BAM files containing reads mapped to the human genome, microbial DNA will be absent, including ancient pathogen DNA that could be highly relevant for an archaeological study. While an ‘unmapped’ BAM ( uBAM) format exists and FASTQ files can be partly reconstructed from such data, raw FASTQ files are the preferred format for data reporting. Unmapped BAM files indicate that a certain level of data preprocessing has already occurred, and such files often combine multiple libraries into a single BAM file in order to achieve sufficient genomic coverage for analysis. If the process of generating the BAM file is not sufficiently described, it can be difficult for other researchers to disentangle the data originating from different libraries or sequencing batches prior to reanalysis. Providing FASTQ files containing merged paired-end reads also limits data reuse. Although read merging is a common first step in some ancient bioinformatics pipelines, it is incompatible with others. Base quality scores are often altered during the read merging process, which can interfere with tools reliant on such scores, and most *de novo* sequence assemblers either require or perform better on unmerged reads. Furthermore, many metagenomic tools, including taxonomic classifiers, do not accept merged paired-end FASTQ files or BAM files as an input format.

*Example:* An ancient metagenomic researcher generates both damage and damage-removed libraries, but merges them together in BAM format and uploads to a sequencing archive. However another researcher wishes to analyse only the data deriving from the damage-removed libraries.

*Solution*: Ideally researchers should upload FASTQ files that match the ‘raw’ output from sequencing, i.e., demultiplexed datasets separated per library, applying only the preprocessing steps recommended by the sequence repository (e.g., for the ENA/SRA, adapter removal but not read merging). If this is not possible, authors should, at a minimum, describe exactly how the merging steps were performed so that other researchers can manually separate merged sequences (e.g., using sequencing read headers) when required for downstream analysis.

**Unique sample accessions applied to multiple libraries of the same sample.**

*Problem:* Another common error was found to occur when researchers mistakenly uploaded each library or sequencing dataset with a unique sample accession code. While often not a critical error, because in these cases the correct sample could usually be inferred from the file name, this nevertheless makes automated data processing more difficult and requires manual intervention. To reuse such data, a researcher must manually reassociate the library datasets with the correct sample based on the file names, rather than relying on the sequence archive sample accession ID, as expected by metadata schemas of the ENA/SRA data repositories.

*Solution:* Researchers should review sequencing archive documentation to ensure they correctly construct upload sheets at both the library and sample levels (e.g. https://ena-docs.readthedocs.io/en/latest/submit/general-guide/metadata.html). Furthermore, researchers should ensure that library names have a consistent pattern such that other researchers can unambiguously associate each library with the correct sample.

### Note on AMDirT generated pipeline scripts

It is important to note that the aim of the pipeline TSV sheets generated by AMDirT is to provide a *template* for data input to the pipelines. Due to the high heterogeneity in the way that sequence (meta) data are uploaded, some information in AncientMetagenomeDir may be missing or erroneous, despite the best efforts of the SPAAM community experts to standardise the information, resolve ambiguities, and correct errors. However, we hope that by providing this functionality, it reduces the time it takes to create such input sheets from scratch.

### Future development

We envision that the future development of the AncientMetagenomeDir project will be to further extend and also standardise the types of metadata currently recorded. For example, when recording the age of samples, AncientMetagenomeDir currently only records a single value of an approximate date. This poses challenges for analyses requiring exact dates and probability intervals such as tip dating for phylogenetic trees and other analyses of evolutionary divergence. At present, however, heterogeneity in the reporting of radiocarbon dates (the most common dating method in archaeology and palaeogenomics) and associated modelling information currently limits our ability to add such dating information to AncientMetagenomeDir and to consistently apply calibration and reservoir effect correction across studies. This is despite the fact that there is already standard reporting guidance. ^21^ However, we also call on ancient metagenomics researchers to report both uncalibrated *and* calibrated dates and associated metadata (radiocarbon lab code, calibration curve, software, etc.), and not to rely solely on secondary citations to facilitate adding such data to repositories such as AncientMetagenomeDir, as well as refinement of chronological modelling in the future.

In the same vein, we also aim to synchronise AncientMetagenomeDir with upstream standardised sequencing data metadata schemas and repositories such as MIxS checklists ^22^ via another SPAAM-established project, MInAS ( https://mixs-minas.org/), to further ensure common standards across both modern and ancient sequencing data.

Given that the functionalities of AMDirT provide simple data exploration without requiring advanced computational knowledge, but also offers semi-prepared templates for aDNA and metagenomics, the dataset and tooling are ideal for further generation of community resources. Following other projects that have been developed for modern microbiome data, ^23^ the ancient metagenomics community could also consider providing standardised and pre-made taxonomic profiles (e.g., for microbiome or environmental samples) or VCF files (for single genomes) that could allow integration into current analysis workflows to assist users in more rapidly integrating public data into their analyses from a single source. This could be particularly useful for screening ancient microbiome samples for preservation (e.g., by comparing a newly sequenced sample to all previously published ancient metagenomes), in order to assess whether a sample falls within the variation of known well-preserved or environmentally degraded samples.

## Conclusions

By extending AncientMetagenomeDir to include library-level metadata, not only do we make ancient metagenomics data more findable, but also we make them more accessible by providing improved transparency of the diverse library and sequencing treatments performed in the field of palaeogenomics. Furthermore, AMDirT has been designed to improve the experience of researchers in the downloading and processing of previously published ancient metagenomics data. By providing both a graphical user interface and a command-line interface to filter and generate relevant download scripts and input sample sheets for aDNA analyses, we provide more flexibility and choice for the wide range of computational backgrounds that ancient metagenomic researchers can have. Finally, we hope that by informing researchers about inconsistencies in past data and metadata uploads and providing templates of standardised metadata for future publications, we will contribute to improving aDNA data reporting and FAIR data sharing.

## Data Availability

The source data for the sample-level metadata used by AMDirT is from the AncientMetagenomeDir project originally published in Ref. 3 under a CC-BY 4.0 license. The existing sample-level and new library-level data is stored on GitHub: https://github.com/SPAAM-community/AncientMetagenomeDir Each release is archived on Zenodo: https://doi.org/10.5281/zenodo.3980833 . New sequencing library-level metadata is also stored in the AncientMetagenomeDir project from version v22.09 (Pyu Ancient Cities) onwards. ^12^ The version of the dataset used for the demonstration of AMDirT, statistics, and figures in this updated manuscript is v24.03 (Monticello). ^24^ Zenodo: SPAAM-community/AncientMetagenomeDir: Monticello (v24.03). https://doi.org/10.5281/zenodo.10942606 . Data are available under the terms of the Creative Commons Attribution 4.0 International license (CC-BY 4.0).

## Software availability

• Software available from:
  – PyPi: https://pypi.org/project/AMDirT/
  – Bioconda: https://bioconda.github.io/recipes/amdirt/README.html
  – Hosted web version of AMDirT viewer: https://spaam-community.org/AMDirT
• Source code available from: https://github.com/SPAAM-community/AMDirT
• Archived source code at time of publication revision (AMDirT v1.6): https://doi.org/10.5281/zenodo.10941007
• License: GNU General Public License v3.0.
