# Supplementary material for: Facilitating accessible, rapid, and appropriate processing of ancient metagenomic data with AMDirT
Source: F1000Res. 2024 May 28;12:926. Originally published 2023 Aug 2. [Version 2] doi: 10.12688/f1000research.134798.2 (PMC11387932; doi:10.12688/f1000research.134798.2)
Supplement: Peer review history

# Reviewer report: Reviewer response for version 2

**DOI:** 10.5256/f1000research.164792.r318837

**Published:** 2024-09-27

**Authors:** Emma Griffiths

**Affiliations:** 1 Simon Frazer University, Burnaby, USA

The manuscript entitled “ Facilitating accessible, rapid, and appropriate processing of ancient metagenomic data with AMDirT“ describes a suite of tools enabling automated population and validation of contextual data (metadata) for samples and libraries in the AncientMetagenomeDir collection. AncientMetagenomeDir is a previously published resource containing sets of tables of curated and standardized metadata for metagenomic and microbial genome datasets generated from ancient samples. AMDirT (AncientMetagenomeDir Toolkit), was developed to facilitate/automate data retrieval and curation from INSDC BioProjects, BioSamples, and SRA/GenBank to support AncientMetagenomeDir. The tooling enabled the addition of library metadata to AncientMetagenomeDir, which is required for many downstream analytical pipelines. Improvements included the addition of a CLI, a GUI, and a web version of AncientMetagenomeDir. Both the GUI and CLI methods can generate standardized templates of metadata, and both a wealth of supporting materials and documentation for users are provided. Code for AMDirT is openly provided on GitHub under a CCBY 4.0 license, and is also available via BioConda. Development of AMDirT was very community-driven, via different hackathons. The developers appear to be highly collaborative (evidenced by the long author and affiliation list acknowledging the efforts of different community members). Improving the quality and findability of metadata and datasets is important, if unfortunately underappreciated, work. AncientMetagenomeDir is a well-curated resource that will likely be very useful to those investigating ancient DNA. Automation of curation and validation is key to sustaining metadata quality and for growing the resource. As such, AMDirT provides important tooling. Furthermore, many use cases and scripts were provided to illustrate how AMDirT can be implemented. The Problem-Solution section in the Discussion was great, and really highlighted issues that aren’t solved by the tooling but could be solved by the adoption of best (or at least better) practices by data providers. 

 To really understand the importance of AMDirT, I had to backtrack through several citations, and review a lot of the AncientMetagenomeDir documentation (click a lot of links). The authors have done their best to be concise and not be redundant in describing the AncientMetagenomeDir as it is already published, but this is sometimes to the detriment of clarity for the reader of the current manuscript. As such, Figure 2 was critical for making sense of AMDirT. Figure 3 was helpful in illustrating important components of the interface. What would have helped in the reading of the current manuscript, would be a table of the fields for samples and libraries so the reader could understand the metadata types that AMDirT was retrieving at-a-glance. 

 Furthermore, an expanded discussion of the need/activities to align AncientMetagenomeDir fields and terms with community standards (i.e. MIxS) that is currently underway, would further demonstrate the authors’ commitment to FAIR data and interoperability. This is important work and should not be glossed over. Further suggestions about the integration of data standards are provided below.

 This manuscript has already been reviewed several times, and the authors have worked hard to improve the software and paper. If the authors expand their discussion of their plans for integrating community standards, then I would recommend this manuscript for indexing.

**Are sufficient details of the code, methods and analysis (if applicable) provided to allow replication of the software development and its use by others?**

 Figure 2 mentions the use of ontologies, but from my investigations, ontologies are not used (there is some controlled vocabulary for the picklist enums for some fields, but these are not ontologies - ontologies use logical relationships to link the controlled vocabulary, and terms are defined and assigned universal identifiers e.g. OBO Foundry ontologies). Are the enums sourced from particular ontologies e.g. ENVO, Uberon? The authors mention that they are participating in another project to implement/further develop a new MIxS package, which is wonderful. It would be great if the authors could expand on that effort in the discussion so the reader doesn’t have to go to so many external sites to learn more, as this seems to be a crucial effort for building interoperability. In addition to this alignment with community standards, the enum controlled vocabulary would benefit from being aligned with community-based ontologies. Ontology terms can be sourced using the EBI Ontology Look-up Service. Furthermore, there has been work in the ontology community to better structure sample material descriptions. For example, there are some enums that precompose concepts that may make them difficult to compare across resources e.g. “skin from leg” precomposes anatomical material (skin) and anatomical parts (leg), which could also be written as “leg skin” as a synonym in other databases, and the precomposition opens the resource up to the “word bomb” problem i.e. you would need lots of terms to capture skin from other parts of the body (skin from arm, skin from foot, skin from knee). Better to separate “material” into different fields e.g. anatomical material, anatomical part, environmental material, environmental site, collection device, collection method. Some work has been done on this by PHA4GE and a similar effort can be seen in the NCBI One Health Enterics package. Separating environmental fields from anatomical fields has also been helpful, and could improve AncientMetagenomeDir annotations. For example, a data provider could include that a Homo sapiens dental calculus (anatomical material) sample was sourced from a desert (environmental site) vs a tomb, which could provide more context for the data.

 Since all of the data is derived from published work, have the authors considered including additional fields for sample processing (e.g. pooling, subsampling), quality control, bioinformatic processing (e.g. dehosting)? There are ontology-based standardized fields available for these methods that may also be informative for users.

Are the conclusions about the tool and its performance adequately supported by the findings presented in the article?

Yes

Is the rationale for developing the new software tool clearly explained?

Yes

Is the description of the software tool technically sound?

Yes

Are sufficient details of the code, methods and analysis (if applicable) provided to allow replication of the software development and its use by others?

Partly

Is sufficient information provided to allow interpretation of the expected output datasets and any results generated using the tool?

Partly

Reviewer Expertise:

data curation, data standards, ontologies, pathogen genomics, data sharing, data interoperability

I confirm that I have read this submission and believe that I have an appropriate level of expertise to confirm that it is of an acceptable scientific standard, however I have significant reservations, as outlined above.

## References

[1] : Facilitating accessible, rapid, and appropriate processing of ancient metagenomic data with AMDirT. *F1000Res* .2023;12: 10.12688/f1000research.134798.2 926 10.12688/f1000research.134798.2 39262445 PMC11387932

# Reviewer report: Reviewer response for version 2

**DOI:** 10.5256/f1000research.164792.r318840

**Published:** 2024-09-10

**Authors:** Intikhab Alam

**Affiliations:** 1 King Abdullah University of Science and Technology, Thuwal, Makkah Province, Saudi Arabia

Borry et al. work entitled "" covers an important topic on metadata of samples. Historically, this is a developing field where initially such data sets were available with minimal metadata. As the field progressed, importance of comprehensive metadata was acknowledged and standards were proposed. However integrating older and new samples with different level of depth in metadata curation is a challenge. This work tries to improve upon such data curation and developed a tool AMDirT with GUI and commandline interface for users to explore metadata.

 Looking at the GUI interface, it appears to have issues when using Firefox browser, the page does not load. Looking at developer tools there are some issues with the javascripts (e.g. stitle.js) saying "not same-origin") and the following details:

 Security Error: Content at https://www.spaam-community.org/AMDirT/ may not load data from https://cdn.jsdelivr.net/npm/@stlite/mountable@0.52.1/build/static/js/8779.995e8fcf.chunk.js.

 However, it appears to work when using google chrome browser, allowing to explore the data and ability to download relevant tables.

 The command line version is difficult to install due to upgrades and other dependencies. It would be great if a version of AMDirT is available for singularity etc.

 Overall, it looks like a very good tool and useful for the community and it is recommended to expand this tool to other types of metagenomes.

Are the conclusions about the tool and its performance adequately supported by the findings presented in the article?

Yes

Is the rationale for developing the new software tool clearly explained?

Yes

Is the description of the software tool technically sound?

Yes

Are sufficient details of the code, methods and analysis (if applicable) provided to allow replication of the software development and its use by others?

Partly

Is sufficient information provided to allow interpretation of the expected output datasets and any results generated using the tool?

Partly

Reviewer Expertise:

Metagenomics of the biosphere

I confirm that I have read this submission and believe that I have an appropriate level of expertise to confirm that it is of an acceptable scientific standard, however I have significant reservations, as outlined above.

# Response

**Published:** 2024-09-11

**Authors:** Maxime Borry

**Affiliations:** Archeogenetics, Max Planck Institute for Evolutionary Anthropology, Leipzig, Germany

Thank you for your positive comments on your manuscript. In regards to the two reservations (using the serverless version in Firefox, and installation)

1. We already explicitly state in the text (Methods > Implementation > Second paragraph), that the streamlit to WebAssembly functionality that is only supported on Chromium-based browsers. This is a known limitation of the Firefox security policies regarding WebAssembly, and unfortunately not something we have influence on for now. We have added a permanent issue in the AMDirT Github repository ( github.com/SPAAM-community/AMDirT/issues/158) to inform users who might be facing this issue.
2. Installation is already possible via pip and conda (both of which handle dependencies). As stated in the README, and the manuscript, we recommend installation in a dedicated environment when using conda. Furthermore, one of the  benefit of having our software on bioconda is the complementary biocontainer project that automatically generates docker and singularity containers for the tool.  You can get a docker version of our tool from quay.io ( https://quay.io/repository/biocontainers/amdirt?tab=tags&tag=latest), and singularity from the galaxy project ( https://depot.galaxyproject.org/singularity/)

We hope these two points address your comments!

 Maxime Borry & James Fellows Yates

# Reviewer report: Reviewer response for version 1

**DOI:** 10.5256/f1000research.147881.r210768

**Published:** 2023-10-18

**Authors:** Jonas Coelho Kasmanas

**Affiliations:** 1 University of São Paulo, São Paulo, Brazil

The submitted manuscript sheds light on an imperative yet often overlooked aspect of current research, specifically metagenomic research: the accessibility and standardization of sample-level metadata, particularly concerning ancient samples. The topic is relevant to the current scientific landscape, especially as the volume of metagenomic sequencing datasets continues to burgeon. With the surge in data, the need for standardized, accessible, and reusable metadata becomes paramount. This manuscript aptly addresses this gap, making it an essential read for researchers in metagenomics, palaeogenomics, and related fields.

 The authors have channeled the collective effort of the research community through hackathon events to update and enhance the 'AncientMetagenomeDir.' Even contacting researchers and actively encouraging them to correct their metadata annotation. Therefore, I understand that this manuscript evolves the AncientMetagenomeDir and provides an additional resource to further increase its accessibility and usability: the AMDirT.

 The introduction of 'AMDirT' as the companion tool to 'AncientMetagenomeDir' is a significant stride forward. By offering functionalities ranging from metadata template generation, and guidance to data validation, the tool exhibits promise in addressing the prevalent challenges in metadata management. Additionally, it helps researchers with less computational background to download samples systematically and gives them access to a GUI. My review goes toward the message delivery, especially during the initial sections of the manuscript.

 During the introduction section, I missed a clear definition of ancient metagenome. This should include a description of what requires the ancient samples to receive special treatment regarding metadata annotation. Naturally, this leads to comparing your effort to past works like the GOLD ( https://doi.org/10.1093/nar/gky977), the MetaSRA  ( https://doi.org/10.1093/bioinformatics/btx334), the gcMeta ( https://doi.org/10.1093/nar/gky1008), or the HumanMetagenomeDB ( https://doi.org/10.1093/nar/gkaa1031).

 I appreciate the extensive AMDirT documentation, including tutorials and videos. However, the manuscript lacks in the description of the tool's workflow and usability. The "Use cases" section of the manuscript makes the tool much clearer. At the same time, it gives the impression that AMDirT is limited to the GUI exploration and template generation guidance. Specifically, in my opinion, the manuscript poorly describes and explores the "Validate" and "Autofill" commands.

 For example, the Validate command does "a variety of checks." It would help if you made it more transparent. Do you have a document detailing the checks and standards? If so, it should be placed here; if not, I would like to see it in the manual. For the Autofill command, I missed a more detailed description of the "improvements" done using R scripts after pulling the metadata from ENA. Specifically, since those commands could be used outside the realm of ancient metagenomic samples. I believe a figure containing the complete schematic workflow from AMDirT would help.

 Regarding the software operation, I missed a storage requirement specification. The paper mentions that the installation requires Python 3.9, but nothing is specified in the GitHub. The installation via pip and conda should include guidance on accessing those tools and creating a specific environment for the AMDirT. Finally, as a minor opinion, have you considered using specialized sample accessing software? For instance, the SRAtoolkit can download samples from the SRA more efficiently than curl.

 If possible, I would like to see a slight reformat in the discussion "Problem/Solutions" section to make it more visible. A simple extra paragraph or bullet points that clearly separate problems and solutions should solve the problem.

**Running and installing the pipeline.**

 I had a relatively smooth experience. However, I kept receiving the following warning upon any usage:

 "WARNING streamlit.runtime.caching.cache_data_api: No runtime found, using MemoryCacheStorageManager"

 Additionally, the convert command did not work. After successfully installing the tool and downloading the AncientMetagenomeDir. I received the following:

 $ AMDirT convert --curl SPAAM-community-AncientMetagenomeDir-60f8a00/ancientmetagenome-hostassociated/libraries/ancientmetagenome-hostassociated_libraries.tsv ancientmetagenome-hostassociated -o ./

 2023-10-17 19:50:32.289 No runtime found, using MemoryCacheStorageManager

 Traceback (most recent call last):

   File "/mnt/tools/miniconda3/envs/amdirt/lib/python3.9/site-packages/streamlit/runtime/caching/storage/in_memory_cache_storage_wrapper.py", line 87, in get

     entry_bytes = self._read_from_mem_cache(key)

   File "/mnt/tools/miniconda3/envs/amdirt/lib/python3.9/site-packages/streamlit/runtime/caching/storage/in_memory_cache_storage_wrapper.py", line 137, in _read_from_mem_cache

     raise CacheStorageKeyNotFoundError("Key not found in mem cache")

 streamlit.runtime.caching.storage.cache_storage_protocol.CacheStorageKeyNotFoundError: Key not found in mem cache

 Traceback (most recent call last):

   File "/mnt/tools/miniconda3/envs/amdirt/lib/python3.9/site-packages/pandas/core/indexes/base.py", line 3790, in get_loc

     return self._engine.get_loc(casted_key)

   File "index.pyx", line 152, in pandas._libs.index.IndexEngine.get_loc

   File "index.pyx", line 181, in pandas._libs.index.IndexEngine.get_loc

   File "pandas/_libs/hashtable_class_helper.pxi", line 7080, in pandas._libs.hashtable.PyObjectHashTable.get_item

   File "pandas/_libs/hashtable_class_helper.pxi", line 7088, in pandas._libs.hashtable.PyObjectHashTable.get_item

 KeyError: 'archive_accession'

   File "/mnt/tools/miniconda3/envs/amdirt/lib/python3.9/site-packages/streamlit/runtime/caching/storage/in_memory_cache_storage_wrapper.py", line 137, in _read_from_mem_cache

     raise CacheStorageKeyNotFoundError("Key not found in mem cache")

 streamlit.runtime.caching.storage.cache_storage_protocol.CacheStorageKeyNotFoundError: Key not found in mem cache

Are the conclusions about the tool and its performance adequately supported by the findings presented in the article?

Yes

Is the rationale for developing the new software tool clearly explained?

Yes

Is the description of the software tool technically sound?

Yes

Are sufficient details of the code, methods and analysis (if applicable) provided to allow replication of the software development and its use by others?

Yes

Is sufficient information provided to allow interpretation of the expected output datasets and any results generated using the tool?

Yes

Reviewer Expertise:

Bioinformatics, metagenome, metadata standardization, machine learning

I confirm that I have read this submission and believe that I have an appropriate level of expertise to confirm that it is of an acceptable scientific standard, however I have significant reservations, as outlined above.

## References

[1] : Genomes OnLine database (GOLD) v.7: updates and new features. *Nucleic Acids Res* .2019;47(D1) : 10.1093/nar/gky977 D649-D659 10.1093/nar/gky977 30357420 PMC6323969

[2] : MetaSRA: normalized human sample-specific metadata for the Sequence Read Archive. *Bioinformatics* .2017;33(18) : 10.1093/bioinformatics/btx334 2914-2923 10.1093/bioinformatics/btx334 28535296 PMC5870770

[3] : gcMeta: a Global Catalogue of Metagenomics platform to support the archiving, standardization and analysis of microbiome data. *Nucleic Acids Res* .2019;47(D1) : 10.1093/nar/gky1008 D637-D648 10.1093/nar/gky1008 30365027 PMC6324004

[4] : HumanMetagenomeDB: a public repository of curated and standardized metadata for human metagenomes. *Nucleic Acids Res* .2021;49(D1) : 10.1093/nar/gkaa1031 D743-D750 10.1093/nar/gkaa1031 33221926 PMC7778935

# Response

**Published:** 2024-05-06

**Authors:** James Fellows Yates

**Affiliations:** Archaeogenetics, Max Planck Instititue for Evolutionary Anthropology, Deutscher Pl. 6, Germany

Thank you for your generally positive review. We apologise for the long time between your review and our revision, this was due to parental leave and extensive travelling of the corresponding authors.

 Our responses to your specific comments are below. Line numbers in our revised manuscript should correspond to the submitted revised word document (required by F1000), once track changes are accepted. Please note depending on which software we opened it, the line numbers were not indicating the same content. The line numbers should correspond to what is opened in Microsoft Office for Mac Word v16.78.3.

**Manuscript Comments**

  

- *During the introduction section, I missed a clear definition of ancient metagenome. This should include a description of what requires the ancient samples to receive special treatment regarding metadata annotation. Naturally, this leads to comparing your effort to past works like the GOLD (* https://doi.org/10.1093/nar/gky977 *), the MetaSRA  (* https://doi.org/10.1093/bioinformatics/btx334 *), the gcMeta (* https://doi.org/10.1093/nar/gky1008 *), or the HumanMetagenomeDB (* https://doi.org/10.1093/nar/gkaa1031 *).* *I appreciate the extensive AMDirT documentation, including tutorials and videos. However, the manuscript lacks in the description of the tool's workflow and usability. The "Use cases" section of the manuscript makes the tool much clearer. At the same time, it gives the impression that AMDirT is limited to the GUI exploration and template generation guidance. Specifically, in my opinion, the manuscript poorly describes and explores the "Validate" and "Autofill" commands.* *For example, the Validate command does "a variety of checks." It would help if you made it more transparent. Do you have a document detailing the checks and standards? If so, it should be placed here; if not, I would like to see it in the manual. *

**Response: **For the ancient metagenome definition: this was originally defined in the AncientMetagenomeDir publication but we’ve added a short sentence rephrasing and citing that definition on line 36-37.

 For validate: we do not go into much detail on this, as it again was originally described in the first AncientMetagenomeDir paper (as cited in the main text) and as such does not necessarily represent ‘new’ functionality other than a renaming. Furthermore, as it is not a user facing component of the tool kit, we feel it is less relevant for readers (who we presume many more will be potential users rather than developers wishing to adopt a similar scheme). However we have rephrased the text slightly to indicate which person each command is most relevant to (e.g. contributor vs maintainer vs user etc.). The same goes for autofill, in that it is not a user facing tool - as it typically will be just within GitHub actions.

 We have however updated the manuscript to provide a little more detail on the validate command (line 105-140) and for autofill (which calls the ENA API for information) in lines 100-103. We have otherwise also expanded the use case to also describe in more detail the ‘convert’ CLI command, which is also a primarily user-facing subcommand.

  

- *For the Autofill command, I missed a more detailed description of the "improvements" done using R scripts after pulling the metadata from ENA. Specifically, since those commands could be used outside the realm of ancient metagenomic samples. I believe a figure containing the complete schematic workflow from AMDirT would help.*

**Response: **We have removed references to the R scripts used in the very initial pulling of data in preparation for the first library-level hackathon. On reflection, indeed this could be confusing to the reader, particularly as these are no longer used - and were only used for a one off event. The same concepts have been added to autofill (which came later). In autofill, the ENA portal API is directly queried using Python. We have added more details on the retrieved metadata fields (line 100-103).

 We have further now reduced the necessity of a user to run this command at all (see above about not being a user-facing tool), instead having a GitHub actions bot to run this command for a user on the AncientMetagenomeDir repository, during a pull request.

 We have included a new figure (Figure 2) that shows the updated AncientMetagenomeDir workflow modified after the original AncientMetagenomeDir publication workflow diagram.

- *Regarding the software operation, I missed a storage requirement specification. The paper mentions that the installation requires Python 3.9, but nothing is specified in the GitHub. The installation via pip and conda should include guidance on accessing those tools and creating a specific environment for the AMDirT. *

**Response: **We unfortunately don’t fully understand what the reviewer means exactly by ‘storage requirement specification’ here. However, based on our interpretation: all downloadable AMDirT files are small bash scripts, TSV, or bibtext files, all of which take up (bioinformatically) negligible amounts of space - single digit megabytes or less. Thus we do not feel it is necessary to mention this. However if the storage requirements refers to FASTQ files etc, as a convenience, AMDirT offers an estimate of the potential total size of the files if you were to run the download script. This is displayed by hovering over the download script download button - however AMDirT does not download FASTQ files itself. This functionality was already described on lines 452.

 The AMDirT package zip archive itself is also less than a megabyte so we feel is not relevant here as it will not make an impact on storage requirements in the vast majority of cases.

 For installation requirements: we are not entirely sure what the reviewer means by  ‘accessing these tools’ - whether this is the downstream dependencies, or the package managers pip/conda themselves. If the former, the version of dependencies management is automatically handled by pip/conda, and is completely transparent for the user as this is reported by each package manager when installing AMDirT. For advanced bioinformaticians, they are likely familiar with python packages setup.py and conda environment files, so should be able to find these themselves (as an everyday user does not need this). If the reviewer is referring to installation instructions of pip and conda, this is a fair point and we have added in the installation instructions URLs to the installation documentation pages of the respective package managers, and we have updated the README to provide updated guidelines to install AMDirT in a dedicated conda environment.

  

- *Finally, as a minor opinion, have you considered using specialized sample accessing software? For instance, the SRAtoolkit can download samples from the SRA more efficiently than curl.*

**Response: **As an alternative to curl, AMDirT already provides download scripts using other tools such as aspera and nf-core/fetchngs , which itself, already, provides the option to download with SRA-tools. We picked these tools based on the interest of AncientMetagenomeDir contributors - of which SRAToolkit was not one at the time, however we have added this option in AMDirT v1.6 at the reviewers request. 

  

- *If possible, I would like to see a slight reformat in the discussion "Problem/Solutions" section to make it more visible. A simple extra paragraph or bullet points that clearly separate problems and solutions should solve the problem.*

**Response: **We are unfortunately somewhat beholden to the formatting of the publisher during typesetting, but we have attempted to reformat the paragraph according to the reviewer’s wish with paragraphs (as each text is too long for a bullet point)

***Running and installing the pipeline.***

- *I had a relatively smooth experience. However, I kept receiving the following warning upon any usage:*

*"WARNING streamlit.runtime.caching.cache_data_api: No runtime found, using MemoryCacheStorageManager"*

 This is unfortunately a known issue resulting from a design decision of one of AMDirT dependencies: the streamlit library and its caching system. As a stop gap solution, we now have ‘ monkeypatched ’ the streamlit function triggering this warning to silence it (https://github.com/SPAAM-community/AMDirT/pull/132).

- *Additionally, the convert command did not work. After successfully installing the tool and downloading the AncientMetagenomeDir. I received the following:*
*$ AMDirT convert --curl SPAAM-community-AncientMetagenomeDir-60f8a00/ancientmetagenome-hostassociated/libraries/ancientmetagenome-hostassociated_libraries.tsv ancientmetagenome-hostassociated -o ./* *2023-10-17 19:50:32.289 No runtime found, using MemoryCacheStorageManager*

*        *


**Response: **The convert command by default takes as input a *sample* table, not a library table, which is already reflected in the documentation of the convert command ( amdirt.readthedocs.io/en/latest/how_to/convert ) and the convert command CLI help.

 ```

 $ AMDirT convert --help

 Usage: AMDirT convert [OPTIONS] SAMPLES TABLE_NAME

   Converts filtered samples and libraries tables to eager, ameta, taxprofiler, and fetchNGS input tables

   Note: When supplying a pre-filtered libraries table with `--libraries`, the

   corresponding sample table is still required!

   SAMPLES: path to filtered AncientMetagenomeDir samples tsv file

   TABLE_NAME: name of table to convert

 Options:

   -t, --tables PATH   (Optional) JSON file listing AncientMetagenomeDir

                        tables

   --libraries FILE    (Optional) Path to a pre-filtered libraries table

                        NOTE: This argument is mutually exclusive with

                        arguments: [librarymetadata].

   --librarymetadata   Generate AncientMetagenomeDir libraries table of all

                        samples in input table NOTE: This argument is

                        mutually exclusive with  arguments: [libraries].

   -o, --output DIRECTORY  conversion output directory  [default: .]

   --bibliography      Generate BibTeX file of all publications in input

                        table

   --curl              Generate bash script with curl-based download

                        commands for all libraries of samples in input table

   --aspera            Generate bash script with Aspera-based download

                        commands for all libraries of samples in input table

   --fetchngs          Convert filtered samples and libraries tables to nf-

                        core/fetchngs input tables

   --sratoolkit        Generate bash script with SRA Toolkit fasterq-dump

                        based download commands for all libraries of samples

                        in input table

   --eager             Convert filtered samples and libraries tables to

                        eager input tables

   --ameta             Convert filtered samples and libraries tables to

                        aMeta input tables

   --mag               Convert filtered samples and libraries tables to nf-

                        core/mag input tables

   --taxprofiler       Convert filtered samples and libraries tables to nf-

                        core/taxprofiler input tables

   --help              Show this message and exit.

 ```

 Thus, for example, running the convert command with a sample table will generate this output:

 ```

 $ AMDirT download -t ancientmetagenome-hostassociated -y samples -r v23.12.0

 $ head -n 10 ancientmetagenome-hostassociated_samples_v23.12.0.tsv > test.tsv

 $ AMDirT convert --curl test.tsv ancientmetagenome-hostassociated

 AMDirT [WARNING]: We provide no warranty to the accuracy of the generated input sheets.

 AMDirT [INFO]: Writing curl download script 

 $ head AncientMetagenomeDir_curl_download_script.sh

 #!/usr/bin/env bash

 curl -L ftp://ftp.sra.ebi.ac.uk/vol1/fastq/SRR132/022/SRR13263122/SRR13263122_2.fastq.gz -o SRR13263122_2.fastq.gz

 curl -L ftp://ftp.sra.ebi.ac.uk/vol1/fastq/SRR132/024/SRR13263124/SRR13263124_2.fastq.gz -o SRR13263124_2.fastq.gz

 curl -L ftp://ftp.sra.ebi.ac.uk/vol1/fastq/SRR132/031/SRR13263131/SRR13263131_2.fastq.gz -o SRR13263131_2.fastq.gz

 curl -L ftp://ftp.sra.ebi.ac.uk/vol1/fastq/SRR132/030/SRR13263130/SRR13263130_2.fastq.gz -o SRR13263130_2.fastq.gz

 curl -L ftp://ftp.sra.ebi.ac.uk/vol1/fastq/SRR957/SRR957740/SRR957740.fastq.gz -o SRR957740.fastq.gz

 curl -L ftp://ftp.sra.ebi.ac.uk/vol1/fastq/SRR957/SRR957744/SRR957744.fastq.gz -o SRR957744.fastq.gz

 curl -L ftp://ftp.sra.ebi.ac.uk/vol1/fastq/SRR957/SRR957741/SRR957741.fastq.gz -o SRR957741.fastq.gz

 curl -L ftp://ftp.sra.ebi.ac.uk/vol1/fastq/SRR957/SRR957738/SRR957738.fastq.gz -o SRR957738.fastq.gz

 curl -L ftp://ftp.sra.ebi.ac.uk/vol1/fastq/SRR132/028/SRR13263128/SRR13263128_2.fastq.gz -o SRR13263128_2.fastq.gz

 ```

 However we’ve added an additional validation check to ensure that the input SAMPLE object is in the valid format expected by the tool, and if not asks the user to check if it is a sample table ( https://github.com/SPAAM-community/AMDirT/pull/140 , commit 7bb6b55)

 We have also added support for supplying *library* level metadata tables (supplied alongside a corresponding samples table) to the convert command with the --libraries parameter. This, alongside the new command download (added since the submission of the original manuscript), allows for an almost end to end command line based interaction with AMDirT: download → (filtering with bash tools) → convert, as reflected in the updated use case section of the manuscript.

# Reviewer report: Reviewer response for version 1

**DOI:** 10.5256/f1000research.147881.r210929

**Published:** 2023-10-09

**Authors:** Timothy Read, Robert Petit III

**Affiliations:** 1 Emory University, Atlanta, Georgia, USA; 2 Wyoming Public Health Laboratory, Cheyenne, Wyoming, USA

This paper describes the ‘AMDirT' open source software for interaction with ancient metagenome sequence sample metadata tables. The tables have been produced through a massive communal curation effort by the international SPAAM (The Standards, Precautions, and Advances in Ancient Metagenomics community) group to systematically improve on what is available from the public repositories of archived short read sequence data.  AMDirT allows viewing and searching of the tables using the Python streamlit library and facilitates download of data and integration with open source nextflow analysis pipelines.  AMDirT also helps with initial creation and validation of new metadata tables.

 I successfully installed AMDirT version 1.4.6 using conda on my Intel MacBook following instructions in the manuscript.  I was able to replicate the commands described. There is also a public server https://www.spaam-community.org/AMDirT/ for the AMDirT viewer. The documentation for the software is very good and includes video tutorials.

 The manuscript is well-written and clearly outlines the functions of the software.  The development of AMDirT represents a significant effort, not least because of the community-wide consultation.  The need for community-based metadata addresses a well-known problem with the SRA/ENA/DDBJ databases and the discussion provides some nice examples of the type of issues faced when downloading.  AMDirT is a valuable tool for both the ancient metagenomics research community and those outside the community interested in browsing and accessing the data.  

**Specific Points**

- The streamlit-based viewer is pretty slick but I feel the importance of command line interface (CLI) is a little underplayed here. The viewer can be used by people without CLI proficiency but to actually take actions like making new sample tables, or download and process data, CLI is essential. 
- The instructions in the text and on github for conda install should guide users to install into a fresh conda environment rather than into the base.
- There should be a list of computational environments that the software has been tested on (e.g do M1-3 macs work?)
- I did not see a mention in the text that there is actually a public facing server https://www.spaam-community.org/AMDirT/ 
- “Newly added library information columns include the library name (how data are typically reported in original publications), the aDNA library generation method (e.g., double-stranded or single-stranded libraries), the library indexing polymerase (e.g., proof-reading or non-proofreading), and the library pretreatment method (e.g., non-Uracil-DNA Glycosylase (UDG), full-UDG, or half-UDG treatments). The latter three fields represent information about the sequencing library construction that influence the presence of aDNA damage, a factor that is critical for the processing of aDNA NGS data.8,18 Sequencing metadata columns include instrument model, library layout (single- or paired-end), library strategy (whole genome sequencing, targeted capture, etc.), and read count. “ I could not work out how to search these fields through the viewer.  These are accessible as downloads post validation but it seems that users would want to search through samples based on these fields?

**Further questions**

 Finally, I have some three questions about the project that it would be great to get comments on.

1. How sustainable is the community effort to maintain these databases moving forward into the future with the probability of the number of samples increasing each year?
2. Have you tried sending the improved metadata back to SRA/ENA?
3. How difficult would it be to adapt the software for a different community of researchers that wanted to improve annotation but use fields that would be different from the ancient metagenomes community?

Are the conclusions about the tool and its performance adequately supported by the findings presented in the article?

Yes

Is the rationale for developing the new software tool clearly explained?

Yes

Is the description of the software tool technically sound?

Yes

Are sufficient details of the code, methods and analysis (if applicable) provided to allow replication of the software development and its use by others?

Yes

Is sufficient information provided to allow interpretation of the expected output datasets and any results generated using the tool?

Yes

Reviewer Expertise:

Bacterial genomics, metagenomics, bacterial genetics, antibiotic resistance

We confirm that we have read this submission and believe that we have an appropriate level of expertise to confirm that it is of an acceptable scientific standard.

# Response

**Published:** 2024-05-06

**Authors:** James Fellows Yates

**Affiliations:** Archaeogenetics, Max Planck Instititue for Evolutionary Anthropology, Deutscher Pl. 6, Germany

Thank you for your positive review. We apologies for the long time between your review and our revision, this was due to parental leave and extensive traveling of the corresponding authors.

 Our responses to your specific points and further questions are as follows. Line numbers should correspond to the submitted revised word document (required by F1000), once track changes are accepted. Please note depending on which software we opened it, the line numbers were not indicating the same content. The line numbers should correspond to what is opened in Microsoft Office for Mac Word v16.78.3.

***Specific Points***

- *The streamlit-based viewer is pretty slick but I feel the importance of command line interface (CLI) is a little underplayed here. The viewer can be used by people without CLI proficiency but to actually take actions like making new sample tables, or download and process data, CLI is essential. *

**Response: **We have made more prominent references to the command line interface functionalities in the manuscript by further specifying in the introduction on lines 75-88, 101-140, and 151-155.

 We also extended the ‘Use case’ section of the manuscript to further guide readers on how they could use AMDirT command line tools to perform the same workflow in the GUI as shown in Figure 3. Note that during the improvement of this use case, we also added an additional command called ‘download’ to further make it easier for CLI power-users to retrieve the tables for a more complete CLI based workflow.
* *

- *The instructions in the text and on github for conda install should guide users to install into a fresh conda environment rather than into the base.*

**Response: ** We have updated the documentation to reflect the new instructions in the code repository README, documentation pages (both can be seen in https://amdirt.readthedocs.io/en/master/README.html ), and the manuscript line 263

- *There should be a list of computational environments that the software has been tested on (e.g do M1-3 macs work?)*

**Response: **We have added a brief section about hardware and OSs that have been tested in the github repository README installation page of the documentation ( https://amdirt.readthedocs.io/en/master/README.html ).

- *I did not see a mention in the text that there is actually a public facing server* https://www.spaam-community.org/AMDirT/

* *

**Response: **There was already a mention of the viewer interface at the line 369, but we have added more details lines 76, and 759


*  *

- *“Newly added library information columns include the library name (how data are typically reported in original publications), the aDNA library generation method (e.g., double-stranded or single-stranded libraries), the library indexing polymerase (e.g., proof-reading or non-proofreading), and the library pretreatment method (e.g., non-Uracil-DNA Glycosylase (UDG), full-UDG, or half-UDG treatments). The latter three fields represent information about the sequencing library construction that influence the presence of aDNA damage, a factor that is critical for the processing of aDNA NGS data.8,18 Sequencing metadata columns include instrument model, library layout (single- or paired-end), library strategy (whole genome sequencing, targeted capture, etc.), and read count. “ I could not work out how to search these fields through the viewer.  These are accessible as downloads post validation but it seems that users would want to search through samples based on these fields?*

**Response: **We have now extended the AMDirT viewer and convert commands to support library level filtering. In the AMDirT viewer this is supported via a secondary table that is loaded after the sample selection has been made by the user. We’ve updated the use case example and Figure 2 to represent these changes.

***Further questions***

*Finally, I have some three questions about the project that it would be great to get comments on.*

- *How sustainable is the community effort to maintain these databases moving forward into the future with the probability of the number of samples increasing each year?*

**Response: **Ancient metagenomics as a field is still very young and therefore has indeed been very manageable in terms of numbers. But of course as it grows it could become more difficult.

 In passive terms - our feeling so far is as we have started as the field is ‘young’ has benefited from the fact we will have good visibility and awareness of the resource within the community, something that would continue to spread as the research area expands through knowledge transfer. With this, we believe that we will have both a) more potential and willing contributors to cope with the increased number of samples and b) as they are already aware, will maybe already format and prepare their own sample and library metadata in a form easy to add to AncientMetagenomeDir. 

 More proactively, we will continue to survey the community to find out what are good motivating factors to continue contributing. We further plan to continue to refine the tooling and automation to make it as easy as possible for contributors to add and review new contributors. The SPAAM community has also recently become affiliated with the ISBA society ( https://isbarch.org ), which can provide financial support for the previously ‘volunteer’ held hackathons. We also have various ideas for new publications that are typically the biggest motivating factor for researchers (e.g. extending the database to include more precise radiocarbon dates which are critical e.g. for phylogenetic dating analyses). Finally we have also expanded our aim of better metadata reporting of ancient DNA samples and sequencing data to a larger project covering the whole of palaeogenomics ( https://www.mixs-minas.org/ ) that will also help further formalise such thinking in the community, and feed-back into the way AncientMetagenomeDir functions, as well as push such standardisation into the INDSC consortium databases (who use MIxS checklists themselves).


*  *

- *Have you tried sending the improved metadata back to SRA/ENA?*

**Response: **We have not (yet) attempted this. We have not actively recorded all the corrections between the reported information in the publications and as changed for the AncientMetagenomeDir repository. These corrections have been also derived from a mixture of ‘obvious’ mistakes in the publication (e.g. single-end sequencing reported, but paired FASTQ files uploaded to the ENA or SRA), to personal communications with the authors of the publication. In some cases the original authors have themselves made the corrections on ENA/SRA themselves. In any case, requesting changes on SRA/ENA would require permission from the original authors of the publications, which would require a large and extensive effort from the volunteers of SPAAM, with likely highly mixed chances of success (students move on, labs have changed/retired etc). In this vein we feel it would be more beneficial to invest time and effort in the MInAS project to correct future submissions at the source rather than historical data.

- *How difficult would it be to adapt the software for a different community of researchers that wanted to improve annotation but use fields that would be different from the ancient metagenomes community?*

**Response: **AMDirT is mostly based around the JSON schemas that define and enforce the structure of the underlying data. Furthermore, because the validate subpackage is designed using a domain-driven design software architecture, the base class holding the different methods responsible for testing the data are generic and can be applied to any dataframe object, as long as they come with their associated JSON schema. In summary, adapting it to a different community would require some modifications (as some part of the code is specific to ancient metagenomics data), but most of AMDirT core functionalities could be adapted without too much effort.
